# Empirical Bayes models for multiple probe type microarrays at the probe level

**DOI:** 10.1186/1471-2105-9-156

**Published:** 2008-03-20

**Authors:** Magnus Åstrand, Petter Mostad, Mats Rudemo

**Affiliations:** 1 Mathematical Sciences, Chalmers University of Technology, and Mathematical Sciences, Göteborg University, S-41296, Göteborg, Sweden

## Abstract

**Background:**

When analyzing microarray data a primary objective is often to find differentially expressed genes. With empirical Bayes and penalized t-tests the sample variances are adjusted towards a global estimate, producing more stable results compared to ordinary t-tests. However, for Affymetrix type data a clear dependency between variability and intensity-level generally exists, even for logged intensities, most clearly for data at the probe level but also for probe-set summarizes such as the MAS5 expression index. As a consequence, adjustment towards a global estimate results in an intensity-level dependent false positive rate.

**Results:**

We propose two new methods for finding differentially expressed genes, Probe level Locally moderated Weighted median-t (PLW) and Locally Moderated Weighted-t (LMW). Both methods use an empirical Bayes model taking the dependency between variability and intensity-level into account. A global covariance matrix is also used allowing for differing variances between arrays as well as array-to-array correlations. PLW is specially designed for Affymetrix type arrays (or other multiple-probe arrays). Instead of making inference on probe-set summaries, comparisons are made separately for each perfect-match probe and are then summarized into one score for the probe-set.

**Conclusion:**

The proposed methods are compared to 14 existing methods using five spike-in data sets. For RMA and GCRMA processed data, PLW has the most accurate ranking of regulated genes in four out of the five data sets, and LMW consistently performs better than all examined moderated t-tests when used on RMA, GCRMA, and MAS5 expression indexes.

## Background

Microarrays are widely used for measuring gene expression in biomedical research. For the purpose of finding differentially expressed genes there exist numerous methods. In early studies genes where often ranked with respect to fold-change. Genes showing fold-change above 2 (or 3) were regarded as potentially regulated and were selected for further investigation. The obvious drawback with such an approach, as pointed out by many authors, is that genes with high fold-change may also be highly variable and thus with low significance of the regulation. On the other hand, since the number of replicates in many studies is small, variance estimators computed solely within genes are not reliable in that very small values can occur just by chance. As a consequence the ordinary t-test suffers from low power and is not a better option for filtering out regulated genes.

Many methods have been proposed to improve on the variance estimator in order to find more powerful statistical tests for differential expression. In empirical Bayes methods [1-8] and the penalized t-test suggested in [9], the gene-specific variance estimator is modified in order to produce more stable results. With proportions determined by the accuracy of the gene-specific variance estimators, a mixture of the gene-specific variance estimator and a global variance estimate is used in place of the gene-specific variance estimator in the denominator of the t-test. Similarly, in the Significance Analysis of Microarrays (SAM) method [10] and the method suggested in [11], a constant is added to the gene-specific sample standard deviation.

Another approach is to pool variance estimators for genes having similar expression level, thus modeling the variance as a function of intensity-level. For example Eaves et al. [12] use a weighted average of the gene-specific variance estimator and a pooled estimate based on the 500 genes with most similar mean expression level, and Jain et al. [13] suggest the local-pooled-error method (LPE) where a variance function fitted to estimated variances and mean intensities is used. Comander et al. [14] pool genes with respect to minimum intensity rather than mean intensity, and Hu et al. [15] use a hierarchical model with a linear relationship between variance and intensity-level. Of these four methods, only the one suggested in [15] takes the accuracy of the gene-specific variance estimators into account when setting the weights for the gene-specific estimator and the pooled estimator, respectively. On the other hand Hu et al. [15] only deal with a linear relationship between variance and intensity-level. A variance to intensity-level dependency is also utilized in the moderated t-test suggested in [6]. The method proposed builds on the moderated t-test suggested in [2,3] with the addition of fitting a loess curve in the scatter plot of logged variance estimators against mean intensity when estimating the model parameters.

The type of arrays considered in this paper is the Affymetrix GeneChip arrays. These arrays are one color arrays and each gene is represented by a set of probes, the probe-set, consisting of 10–16 probe-pairs. Each probe-pair consists of one perfect match (PM) probe and one mismatch (MM) probe. The probes are 25 bases long and the PM and MM probes have identical sequences of bases except for the middle probe which in the MM probe is set to the complementary base of that in the PM probe. The MM probes are thus designed to measure the background intensity for the corresponding PM probe. The standard way of dealing with the multiple-probes is to derive a probe set summary, an expression index, for each probe-set (gene) and array (sample), for example using the RMA method [16] or the Affymetrix MAS5 algorithm. The expression indexes are then used in downstream analysis by only considering the expression index itself, the precision of the expression index is ignored. However, in the fully Bayesian probe-level BGX model [17] information about the accuracy of the expression index is obtained as a complete distribution which is subsequently used when computing the posterior distribution of differential expression. Also, the probe-level measurement error from the probabilistic probe-level model multi-mgMOS [18] is used when computing the probability of positive log-ratio in the PPLR method [19].

**Figure 1:**
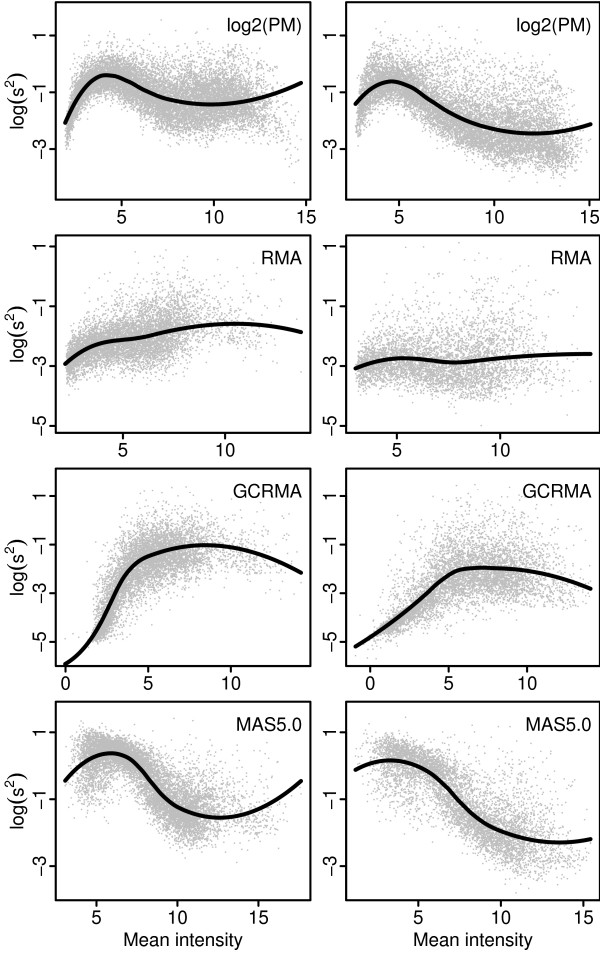
**Probe-, or probe-set, wise sample variances against sample means**. Scatter plots of sample variance *s*^2 ^(logged with base 2) against mean intensity for logged PM intensities and three expression indexes. Left and right panels show data set A and B, respectively (see Section *Data sets*).

**Figure 2:**
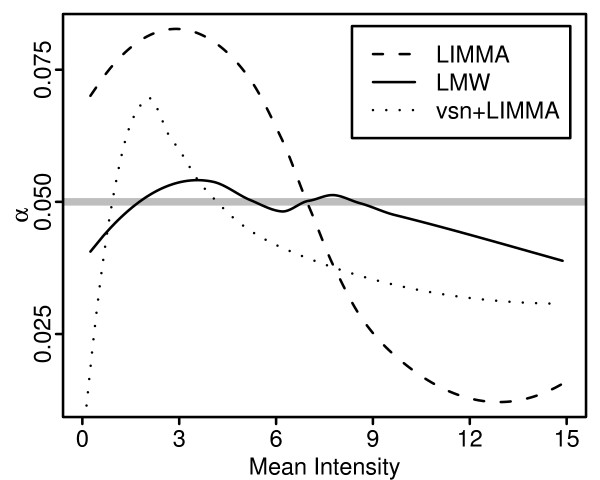
**False positive rate against mean intensity**. False positive rate (*α*) calculated on re-sampled data and plotted against mean intensity. 100 data sets of size 6 were sampled from the complete data set B (see Section *Data sets*) of 18 replicated arrays and then analyzed using the Affymetrix MAS5 algorithm followed by a two group analysis of 3+3 arrays using the moderated t-test in the R-package LIMMA [3], on logged MAS5 indexes and indexes transformed using the variance stabilizing transformation in the R-package vsn [21], and the proposed method LMW using logged MAS5 indexes. false positive rate were obtained by averaging over the sampled data sets using loess-curves fitted to mean intensity and indicator of significance (1 if the probe-set is among the 5% probe-sets with highest absolute statistic, 0 otherwise). The mean intensities of each data set are shifted to the range [0,15].

For Affymetrix type arrays a dependency between variability and intensity-level generally exists, even for log-transformed data. Figure 1 shows scatter plots of sample variance versus sample mean calculated on logged PM intensities (background corrected and normalized using the default methods of RMA) and three different expression indexes: RMA, GCRMA and MAS5. Except for the RMA expression index a clear dependency between variability and intensity-level exists, with a unique signature for each type of pre-processing of the raw CEL-file data. The GCRMA expression index shows increasing variability with intensity-level while MAS5 shows the opposite relationship. As a consequence, methods assuming constant variance as well as methods adjusting the gene-specific variance (or standard deviation) estimators towards a global estimate suffer from intensity-level dependent false positive rates. Figure 2 shows an example where the moderated t-test in the R-package LIMMA [3] was used on MAS5 expression indexes computed on a set of replicated arrays. The false positive rate obtained with LIMMA follows the same pattern as in the right lower panel in Figure 1 where the same data set is used. Almost identical result was obtained using data set A in a similar simulation (data not shown).

The aim of variance stabilizing transformations is to reduce or eliminate the problem of dependency between variability and intensity-level. A family of transformations, the generalized-log family (glog), was introduced in [20-22] and further used in [23,24]. A comparison of the glog family with the started logarithm transformation [25] and the log-linear hybrid transformation [26] is presented in [27]. It is concluded that the glog family is "probably the best choice when it is convenient to use it", but it is also noted that the direct interpretation of differences as logged ratios for microarray data when using the ordinary log-transformation, does not hold when using such variance stabilizing transformations.

Generally, the glog family effectively stabilizes the variance when applied to raw Affymetrix probe-level data, for example using the parameter estimation procedure described in [21]. However, the transformations implicitly defines a background correction, and when applied to data already having been subject to another background correction (or further processed data), the glog transformations may not be able to capture the structure of the dependency between variability and intensity-level. This applies, for example, to probe-level data background corrected using the RMA default background method, and MAS5 expression indexes, see Figure 2. Thus, there is a need for more flexible solutions, and in short, Figures 1 and 2 may be seen as one motivation for the methods proposed in this paper.

The hierarchical Bayesian model WAME proposed and developed in [4,5,7,8] is in the present paper extended to incorporate the variability to intensity-level dependency. The Probe level Locally moderated Weighted median-t method (PLW) applies the extended model to logged PM intensities resulting in moderated and weighted t-statistics for all PM probes. In the final step of PLW the median t-statistic of all PM probes building up each probe-set is computed, and this median is the value used for ranking the probe-sets with respect to differential expression.

The Locally Moderated Weighted-t method (LMW) is a more general method intended for single probe type of arrays or summary measures of multiple probe type arrays, such as RMA and MAS5. LMW use the same model as PLW but since only one t-statistic is obtained for each probe-set no median is calculated. The proposed methods are compared with existing methods on five publicly available spike-in data sets.

## Results and Discussion

### Model and methodology

Given a set of *n *arrays let *y*_*ip *_be the background corrected and normalized log-intensity on array *i *for PM probe *p *and put *y*_*p *_= (*y*_1*p*_,...,*y*_*np*_)^*T*^. The PM probes are divided into *G *(disjoint) probe-sets $G_{1},...,G_{G}$ and thus there are a total of $P=\left|G_{1}\right|+\cdots +\left|G_{G}\right|$ probes. For *p *= 1,...,*P *assume

(1) $$\begin{array}{l} y_{p}|c_{p}\sim {\text{N}}_{n}(\mu_{p},c_{p}\Sigma )\\ c_{p}\sim \Gamma^{-1}(\frac{1}{2}m,\frac{1}{2}m\cdot \nu ({\bar{\mu }}_{p})) \end{array}$$

where *μ*_*p *_is the log-intensity profile for probe *p *across the *n *arrays with mean log-intensity level ${\bar{\mu }}_{p}$, ∑ is an *n × n *covariance matrix, *m *is a real-valued parameter, and *ν*(·) is a smooth real-valued function. N_*n *_denotes an *n*-dimensional normal distribution, and Γ^-1^(*a, b*) denotes the inverse-gamma distribution with shape parameter *a *and scale parameter *b*. A cubic spline is used to parameterize the function *ν*(·). Given set of *K *interior spline-knots

*ν*(*x*) = exp{*H*(*x*)^*T*^*β*}

where *β *is a parameter vector of length 2*K *- 1 and *H *: ℝ → ℝ^2*K*-1 ^is a set of B-spline basis functions, see chapter 5 of [28].

As in the model suggested in [4] the model in Equ. 1 makes use of a global covariance matrix, thus allowing differing variances as well as correlations between arrays. To account for the dependency between variability and intensity-level the scale-parameter of the Γ^-1^-distribution depends on the mean log-intensity level ${\bar{\mu }}_{p}$ for the probe through the smooth function *ν*.

We assume that the vector *μ*_*p *_is determined by a full rank *n × k *design matrix *D *and a parameter vector *γ*_*p *_of length *k*. The aim is to estimate and test hypothesis for *δ*_*p*_, a linear combination of *γ*_*p *_specified by a 1 × *k *matrix *C*. In summary,

*μ*_*p *_= *Dγ*_*p *_and *δ*_*p *_= *Cγ*_*p*_.

For the special case of comparing two conditions, with *n*_1 _and *n*_2 _arrays from conditions 1 and 2, respectively, the design matrix *D *is an (*n*_1 _+ *n*_2_) × 2 matrix. For example, with *n*_1 _= 3 and *n*_2 _= 4 we can use

$$D^{T}=\left[\begin{array}{ccccccc} 1 & 1 & 1 & 0 & 0 & 0 & 0\\ 0 & 0 & 0 & 1 & 1 & 1 & 1 \end{array}\right]\text{ and }\gamma_{p}=\left[\begin{array}{c} \gamma_{p1}\\ \gamma_{p2} \end{array}\right]$$

and thus *μ*_*p *_= (*γ*_*p*1_, *γ*_*p*1_, *γ *_*p*1_, *γ*_*p*2_, *γ*_*p*2_, *γ*_*p*2_, *γ*_*p*2_)^*T*^. With *C *= [-1 1] we have *δ*_*p *_= *γ*_*p*2 _- *γ*_*p*1_, thus *δ*_*p *_is the logged fold change between conditions 2 and 1.

However, instead of estimating the parameters of the model in Equ. 1 we use a reduced model derived from Equ. 1 through a linear transformation of the vector *y*_*p*_. Define the *n × n *and *n *× 1 matrices

*A*_0 _= *I - D*(*D*^*T *^*D*)^-1 ^*D*^*T *^and *B *= *D*(*D*^*T *^*D*)^-1^*C*^*T*^.

Since *A*_0 _is of rank *n - k *only we let *A *be an *n *× (*n - k*) matrix whose column space equals that of *A*_0_.

With *q *= *n - k *+ 1 form the *n × q *transformation matrix *M *and the vector *z*_*p *_of length *q*

(2) *M *= [*A*; *B*] and *z*_*p *_= *M*^*T *^*y*_*p*_

giving the reduced model

(3) $$\begin{array}{l} z_{p}|c_{p}\sim {\text{N}}_{q}\left({\left(0,...,0,\delta_{p}\right)}^{T},c_{p}\Sigma_{z}\right)\\ c_{p}\sim \Gamma^{-1}(\frac{1}{2}m,\frac{1}{2}m\cdot \nu ({\bar{\mu }}_{p})) \end{array}$$

where ∑_*z *_= *M*^*T*^∑*M*.

The reduced model is fitted using the EM algorithm [29] as described in Section *Parameter estimation*.

The *c*_*p*_'s are treated as missing data and we replace the unknown intensity-level for probe *p*, ${\bar{\mu }}_{p}$, with the observed mean intensity across arrays, ${\bar{y}}_{p}$. Given estimators of the parameters ∑_*z*_, *m*, and *β *we proceed as if these parameters are known, and weighted moderated t-tests are computed for each probe *p*. The unbiased minimum variance estimator of *δ*_*p *_is

(4) $${\hat{\delta }}_{p}{\left(\lambda^{T}\Sigma_{z}^{-1}\lambda \right)}^{-1}\lambda^{T}\Sigma_{z}^{-1}z_{p}$$

where *λ *is the vector (0,...,0, 1)^*T *^of length *q*. The weighted moderated *t*-statistic is defined as

(5) $${\tilde{t}}_{p}=\sqrt{\frac{q+m-1}{{\left(\gamma^{T}\Sigma_{z}^{-1}\lambda \right)}^{-1}}}\frac{{\hat{\delta }}_{p}}{\sqrt{m\exp\{H{\left({\bar{y}}_{p}\right)}^{T}\beta \}+{\text{RSS}}_{p}}}$$

and under H_0_: *δ*_*p *_= 0 it can be shown that ${\tilde{t}}_{p}$ is *t*-distributed with *q *+ *m *- 1 degrees of freedom. Here

(6) $${\text{RSS}}_{p}=z_{p}^{T}\left(\Sigma^{-1}-\Sigma^{-1}\lambda {\left(\lambda^{T}\Sigma^{-1}\lambda \right)}^{-1}\lambda^{T}\Sigma^{-1}\right)z_{p}$$

is the weighted residual sum of squares. See [5] for details. The PLW statistic for the probe-set G is then defined as

(7) $${\text{PLW}}_{G}=\text{median}\left\{{\tilde{t}}_{p}:p\in G\right\}.$$

The LMW and PLW methods are implemented in the R package plw [30] available at the authors' web page and at the Bioconductor projects web page [31] (at the time of writing only among devel-packages, bioconductor 2.2).

### Parameter estimation

The *q × q *covariance matrix ∑_*z *_of the reduced model in Equ. 3 is divided according to

$$\Sigma_{z}=\left[\begin{array}{cc} \Sigma_{A} & \Sigma_{AB}\\ \Sigma_{AB}^{T} & \sigma_{B}^{2} \end{array}\right]$$

where ∑_*A *_is the covariance matrix for all but the last dimension of *z*_*p *_and $\sigma_{B}^{2}$ is the variance of the last dimension (indexes *A *and *B *refer to the corresponding sub-matrices of the transformation matrix *M *in Equ. 2). The reduced model is fitted in two steps. First the parameters *m*, *β *and the sub-matrix ∑_*A *_are estimated by dropping the last dimension of the vectors *z*_*p*_. Since the reduced model is not identifiable without a restriction on the function *ν *or the covariance matrices ∑_*z *_we use the restriction trace(∑_*A*_) = *q *- 1. Secondly, the parameters *m *and *β *are held fixed and ∑_*z *_is estimated using the complete *z*_*p *_vectors. Temporarily the assumption of no regulated genes is used (*δ*_*p *_= 0 for all probes) and ∑_*z *_is estimated under the restriction that the trace of the ∑_*A *_part should be equal to *q *- 1.

In step 1, we let *x*_*p *_denote the sub-vector of *z*_*p *_obtained by dropping the last element. Under the reduced model *x*_*p *_is distributed according to the model in Equ. 1 with ∑ = ∑_*A*_, *μ*_*p *_= 0, *n *= *q *- 1, and using the EM-algorithm an iterative procedure for estimating *m*, *β *and ∑_*A *_is obtained. Given estimates of the previous iteration, *m*_0_, *β*_0 _and ∑_*A*0_, updated estimates are found as follows. Let

$$w_{p}=\frac{m_{0}+q-1}{x_{p}^{T}\Sigma_{A0}^{-1}x_{p}+m_{0}\exp\{H{\left({\bar{y}}_{p}\right)}^{T}\beta_{0}\}}.$$

The updated estimate of ∑_*A *_is

(8) $${\hat{\Sigma }}_{A}=\frac{1}{P}\sum_{p=1}^{P}w_{p}x_{p}x_{p}^{T}$$

and the updated estimate of *β *is found by numerical maximization of the function

(9) $$h(\beta )=\frac{1}{P}\sum_{p=1}^{P}\left(H{\left({\bar{y}}_{p}\right)}^{T}\beta -w_{p}\exp\{H{\left({\bar{y}}_{p}\right)}^{T}\beta \}\right).$$

With $\hat{\beta }$ equal to the updated estimate of *β *let

$$\begin{array}{c} S=h(\hat{\beta })-\log(m_{0}+q-1)\\ +\psi \left(\frac{m_{0}+q-1}{2}\right)+\frac{1}{P}\sum_{p=1}^{P}\log(w_{p}) \end{array}$$

where $\psi (x)=\frac{d}{dx}\log\Gamma (x)$ is the digamma function. The updated estimate of *m *is then found using numerical maximization of the function

(10) *f*(*m*) = *m *(log(*m*) + *S*) -2 log (Γ(*m*/2)).

In step 2 a similar iterative procedure is used to estimate ∑_*z*_. With ∑_*z*0 _denoting the estimate of ∑_*z *_from the previous iteration and with *w*_*p *_re-defined as

$$w_{p}=\frac{\hat{m}+q}{z_{p}^{T}\Sigma_{z0}^{-1}z_{p}+\hat{m}\exp\{H{\left({\bar{y}}_{p}\right)}^{T}\hat{\beta }\}}$$

where $\hat{m}$ and $\hat{\beta }$ are the estimates obtained in step 1, an updated estimate of ∑_*z *_is computed according to Equ. 8 with *z*_*p *_replacing *x*_*p*_. In order for the estimators of ∑_*A *_and ∑_*z*_, in step 1 and 2, respectively, to comply with the trace restriction the updated estimates are scaled at the end of each iteration. [For more details see Additional file 1]

### Data sets

The two data sets used in Figures 1 and 2 are publicly available at the Gene Expression Omnibus repository [32] with series or sample reference number indicated below. Data set A consists of the 18 arrays from the severe group of the COPD data set [33] (series reference number GSE1650), where Affymetrix arrays of type HG U133A were used. In data set B the 18 arrays with normal tissue where selected from a lung tumor data set [34] (sample reference numbers GSM47958-GSM47976, excluding GSM47967). Here the HG-U95A arrays were used.

Five spike-in data sets were used to evaluate the proposed methods. In the Affymetrix U95 and 133A Latin Square data sets [35] arrays of type HG-U95A and HG-U133A, respectively, were used. The Affymetrix U95 data set consists of data from 59 arrays divided into 19 groups of size 3, and one group of size 2.

From the 20 groups there are 178 possible pair-wise group comparisons each with 16 [36] known differentially expressed genes among the 12626 genes present on the arrays. The Affymetrix 133A data set comprise data from 42 arrays with a total of 22300 probe-sets of which 42 were spiked in at known concentration. The 42 arrays are divided into 14 groups of size 3 and thus there are 91 possible pair-wise group comparisons. As done in the Affycomp II assessments [36] we exclude 271 probe-sets which are likely to cross-hybridize to spike-in probe-sets. The sequence of each spike-in clone was blasted against all HG-U133A target sequences (~600 bp regions from which probes are selected). A threshold of 100 bp identified 271 probe-sets which are available in the Affycomp R-package.

From the Gene Logic Tonsil and AML data sets [37] all groups with 3 replicated arrays were used, giving a total of 12 and 10 groups, respectively. For these data there are 11 genes spiked in at known concentration, which can be studied in 66 and 45 pair-wise group comparisons, respectively. Both data sets were obtained using the Affymetrix HG-U95A arrays having 12626 genes.

The Golden Spike data set [38] consists of 6 arrays of type Drosgenome1 divided into 2 groups of equal size. The samples used in this experiment consist of mRNA from 3866 genes, of which 1331 are differentially expressed between the groups. The Drosgenome1 array has a total of 14010 genes, thus 10144 of these should not be expressed, 2535 should be expressed but not regulated, and 1331 should be expressed and regulated.

Since all 1331 genes are up-regulated in the spike-in group it is necessary to take special care in the normalization when analyzing this data set. Generally this means performing normalizations based on a subset of genes, either only the 2535 genes spiked in at identical concentration in both groups [39], or the 2535 genes together with the 10144 absent genes [38]. Thus, knowledge about which genes are regulated, which of course is not available for a real situation, is used in the normalization.

For PPLR, BGX, and the analysis based on MAS5 expression indexes we used a loess-subset normalization of probe set summaries as done in [17,19,38]. For the analysis based on RMA (and GCRMA) pre-processed data we used a loess-subset normalization of PM probe intensities similar to the one performed in [40]. PM probe intensities were corrected for background using the default background method of RMA (or GCRMA) and then loess-normalized using the same subset as used in [38], thus the 2535 genes spiked in at identical concentration in both groups together with the 10144 absent genes. At this point PLW, median-t, and combined-p were applied to logged PM intensities. Probe set summaries using median polish were then computed and all 10 methods using expression indexes as input were used to rank genes with respect to differential expression.

### Comparison with existing methods

Using the spike-in data sets listed above the proposed methods, PLW and LMW were compared with 14 existing methods for ranking genes. The 14 methods include ranking with respect to: observed fold change (FC), ordinary t-test, the moderated t-test in the R-package LIMMA [3], the weighted moderated t-test in the R-package WAME.EM [8], Efron's penalized t-test [11] and the Shrink-t method [9] in the R-package st, the SAM method [10] in the R-package samr, the Local-pooled-error test [13] in the R-package LPE, the Intensity-Based Moderated T-statistic (IBMT) [6] using the R-code available at , and the two probe-level methods median-t and combined-p suggested by Hess and Iyer [40].

All methods using expression indexes as input (including PLW) were applied to RMA, GCRMA and MAS5 expression indexes obtained using the R-package affy, while PLW, median-t, and combined-p were applied to logged PM intensities, background corrected and normalized using the default methods of RMA and GCRMA. (The empirical Bayes approach of GCRMA was used to calculate background corrected intensities, thus the fast option was set to FALSE). With LMW 4–6 spline-knots (depending on the number of probe-sets) were used for the function *ν*, whereas 12 knots were used in PLW (the spline-knots are set using an internal function in the R-package plw). Note that RMA and GCRMA were applied only to the arrays involved in each group comparison, as opposed to running RMA and GCRMA using all arrays of each data set.

We also compared with the PPLR method [19] applied to the expression index and probe-level measurement error of the multi-mgMOS model [18] available in the R-package puma, the logit-t procedure implemented in the R-package plw according to the description in [41], and the BGX method [17] as implemented in the R-package bgx. The R-code used for each of the 14 methods is available as supplementary material. [See Additional file 2]

Due to long computer run times the comparison with the BGX method is restricted to the Golden Spike data set, and a subset of genes from the Gene Logic AML data set (the run time for one single analysis of 6 arrays with all 12626 probe-sets is more than 24 hours). The subset of size 1011 of the Gene Logic AML data set consists of probe-sets number 6000–7002 (excluding 6030, 6367, and 6463) together with the 11 spiked probe-sets and the same subset was used in [17]. The probe-set numbering is as obtained when loading data into R using the R-package affy. Also, to avoid introducing yet another normalization of the Golden Spike data set, logit-T was not applied to this data set.

For each spike-in data set and combination of method and expression index/pre-processing ROC-curves were calculated. Also, for the analysis using a complete set of probe-sets, the area (AUC) under the ROC curve up to 25, 50, 100 and 200 false positives was computed. In the comparison with BGX using only 1011 probe-sets, AUC was computed up to 2, 4, 8 and 16 false positives in order to cover the same false positive range as for the complete probe-set comparisons.

**Figure 3:**
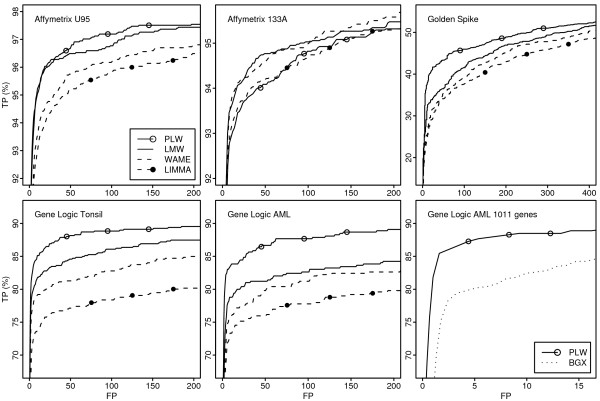
**ROC curves**. ROC curves for a subset of the methods compared when applied to RMA pre-processed data. The horizontal axis shows the number of false positives (FP) and the vertical axis the proportion of true positives found (TP).

For the analysis based on RMA pre-processing, ROC curves for a subset of the compared methods are found in Figure 3. AUC values up to 100 false positives from the complete probe-set analysis are found in Tables 1 and 2. (ROC curves for all methods and AUC up to 25, 50, 200 false positives are available as supplementary material. [See Additional file 3 and Additional file 4])

**Table 1:** Area under ROC curves up to 100 false positives, RMA and GCRMA

Method	Pre-processing	Affymetrix U95	Affymetrix 133A	Golden Spike	Gene Logic Tonsil	Gene Logic AML
PLW	RMA	96(1)	93(6)	42(1)	87(1)	86(1)
LMW	RMA	96(2)	94(1)	36(5)	84(3)	80(5)
LPE	RMA	94(7)	93(11)	40(2)	84(2)	85(2)
combined-p	RMA	95(4)	92(12)	39(3)	83(4)	81(4)
WAME	RMA	95(5)	94(2)	33(7)	81(7)	78(8)
median-t	RMA	95(3)	93(10)	39(4)	82(6)	80(6)
IBMT	RMA	95(6)	94(3)	34(6)	78(9)	76(9)
Efron-t	RMA	94(8)	93(4)	32(8)	79(8)	79(7)
FC	RMA	92(12)	93(5)	29(12)	83(5)	85(3)
LIMMA	RMA	94(9)	93(7)	32(9)	76(10)	75(10)
SAM	RMA	94(10)	93(8)	32(11)	74(12)	74(11)
Shrink-t	RMA	94(11)	93(9)	32(10)	75(11)	73(12)
t-test	RMA	85(13)	86(13)	21(13)	57(13)	52(13)

PLW	GCRMA	97(1)	92(8)	54(1)	87(1)	87(1)
LMW	GCRMA	95(3)	93(1)	50(5)	84(3)	79(6)
median-t	GCRMA	96(2)	92(10)	50(2)	83(4)	81(5)
combined-p	GCRMA	95(5)	91(12)	50(4)	86(2)	81(4)
LPE	GCRMA	95(6)	91(11)	50(3)	82(6)	86(2)
IBMT	GCRMA	95(4)	93(2)	47(6)	81(9)	76(8)
Efron-t	GCRMA	94(7)	93(4)	37(8)	82(7)	79(7)
WAME	GCRMA	94(10)	93(3)	39(7)	83(5)	75(10)
FC	GCRMA	93(12)	93(7)	30(13)	81(8)	86(3)
LIMMA	GCRMA	94(9)	93(5)	37(9)	80(10)	73(11)
SAM	GCRMA	94(8)	93(6)	36(11)	79(11)	76(9)
Shrink-t	GCRMA	94(11)	92(9)	37(10)	78(12)	70(12)
t-test	GCRMA	86(13)	84(13)	30(12)	64(13)	53(13)

Area under ROC curves up to 100 false positives rounded to nearest integer value with an optimum of 100. Numbers within parenthesis are within data set ranks for the methods compared (separately for RMA and GCRMA). Methods are ordered with respect to mean rank across the five data sets. Results in the upper and lower part are based on RMA and GCRMA pre-processed data, respectively.

**Table 2:** Area under ROC curves up to 100 false positives, MAS5, PPLR, BGX, and Logit-T

Method	Pre-processing	Affymetrix U95	Affymetrix 133A	Golden Spike	Gene Logic Tonsil	Gene Logic AML
LMW	MAS5	89(1)	87(1)	60(1)	79(1)	70(2)
IBMT	MAS5	87(2)	87(2)	59(2)	77(3)	69(3)
LPE	MAS5	84(3)	84(3)	57(3)	78(2)	79(1)
WAME	MAS5	71(6)	81(5)	34(5)	69(4)	54(8)
SAM	MAS5	74(4)	81(4)	11(8)	67(5)	54(9)
LIMMA	MAS5	71(7)	81(6)	31(6)	67(6)	54(6)
Shrink-t	MAS5	71(8)	80(7)	23(7)	67(7)	54(7)
t-test	MAS5	73(5)	76(8)	39(4)	60(9)	47(10)
Efron-t	MAS5	65(9)	72(9)	3(9)	66(8)	57(4)
FC	MAS5	56(10)	61(10)	0(10)	58(10)	55(5)

BGX		-	-	58	-	75*
logit-T		94	92	-	80	79
PPLR	multi-mgMOS	88	90	57	71	69

# of genes		12626	22029	14010	12626	12626
# of spikes		16	42	1331	11	11
# of groups		20	14	2	12	10

Area under ROC curves up to 100 false positives rounded to nearest integer value with an optimum of 100. Numbers within parenthesis are within data set ranks for the methods compared, and methods are ordered with respect to mean rank across the five data sets (within MAS5 only). *Results in *italic *are from the subset of 1011 probe sets of the Gene Logic AML data set.

Results for RMA pre-processing are found in the upper part of Table 1. Three of the four methods taking the variability-to-intensity-level dependency into account (PLW, LMW and LPE) performed overall better than the other methods, with the proposed method PLW having the highest AUC on four of the five data sets. The fourth method taking the variability-to-intensity-level dependency into account (IBMT) performed comparably well on the Affymetrix and Golden Spike data sets but less so on the two Gene Logic data sets.

Ranking genes with respect to FC performs quite well on the Affymetrix U133A and the two Gene Logic data sets but not on the other two data sets. Among the penalized and moderated t-test methods, WAME and Efron-t consistently perform better than the other ones. However, the difference between these methods for the two Affymetrix Latin Square and the Golden Spike data sets are small, compared to the difference in AUC obtained using the two Gene Logic data sets. Thus, the two Gene Logic data sets appear slightly different from the other three.

The results obtained using GCRMA in the lower part of Table 1 are very similar to the results with RMA shown in the upper part. For the Golden Spike data set, replacing GCRMA with RMA improves the performance of all methods but the ordering of the methods is fairly unchanged. Overall, IBMT, Efron-t and median-t, performs slightly better when applied to GCRMA expression indexes whereas WAME and LPE performs slightly worse. The overall ordering of the top-two methods is unchanged.

Since MAS5 expression indexes show a very clear dependency between variability and intensity level in Figure 1, and since the variability decreases with intensity it comes as no surprise that all three methods taking this dependency into account consistently performs better than all other methods as shown in the upper part of Table 2. The LMW method has the most accurate ranking of genes in 4 out of the 5 data-sets, and performs better than the IBMT method on all 5 data-sets. Since the main difference between LMW and IBMT is that LMW performs a weighted analysis, and since WAME overall performs better than LIMMA, it appears as if weighted analysis should be used in preference to analysis using unweighted analysis.

The lower part of Table 2 shows results for PPLR, BGX, and logit-t. For the Golden spike data set both PPLR and BGX perform comparably well, only LMW and IBMT applied on MAS5 expression indexes have higher ROC curve AUC. In the analysis of 1011 probe sets from the Gene Logic AML data set, PLW shows consistently higher true positive rate compared with BGX (Figure 3) and the AUC up to 8 false positives (scaled so that optimum is 100) is 84 and 75 for PLW and BGX, respectively. For the remaining data sets PPLR performed comparably well with other methods using MAS5 expression indexes, but less so when comparing with RMA and GCRMA pre-processed data.

The second proposed method LMW differs from existing moderated and penalized t-test in that the global variance estimator (which gene-specific estimators are adjusted towards) varies with intensity-level.

Actually this is the only difference between LMW and the WAME method. The LPE method also uses a global variance estimator that varies with intensity-level. But opposed to using a weighted mean of the global and gene-specific estimator, only the global estimator is used in the denominator of the LPE statistic. Thus for genes with similar intensity-level, LPE is basically identical to ranking using fold change. Hence, since LMW consistently performs better than WAME, and LPE has higher AUC than fold change in four of the five data test, modeling the global variance estimator as a function of intensity is worthwhile doing.

Figure 2 shows that the false positive rate obtained by adjusting towards a global estimate that varies with intensity-level results in a much more stable false positive rates compared to using a (truly) global estimate. It should be mentioned that as the number of arrays increases, the variability of the false positive rate across intensity-level decreases, when adjusting towards a truly global estimate as well as when adjusting towards an intensity-level dependent global estimate. Figure 2 is based on 3+3 arrays, and the estimated false positive rate for LIMMA varies between 1.3% and 8.3%. When repeating the same analysis using 5+5 arrays the estimated false positive rate varied between 1.8% and 7%.

With RMA (and GCRMA) pre-processed data, we do comparisons between 1) Compute probe set summaries from probe intensities and then do inference, and 2) Do inference for each PM probe and then summarize into one score. Thus, the summarization (here done using median-polish) is considered as a part of the first approach. PLW and median-t use the second approach, whereas LMW and the ordinary t-test are the corresponding methods using the first approach. From the results presented here the second approach appears a better option. This was also shown by Hess and Iyer in [40] where they propose the median-t and combined-p method.

Approach 1 could also include a second normalization of probe set summaries. However, neither of the two approaches can be given information about which genes that are regulated without making the comparison biased. Thus, in the analysis of the Golden Spike data set, a second subset-loess normalization of probe set summaries as done in [38] can not be used when comparing approach 1 and 2. We therefore used a subset-loess normalization of PM probe intensities in a similar way as done in [40].

We have also computed results for the Golden Spike data set using subset-loess normalized MAS5 expression indexes as done by Choe et al. in [38]. They show that, for a large set of different pre-processing methods, a second loess-subset normalization of probe-set summaries has a large effect (Figure 7a). They give no direct answer to whether a subset-based probe normalization to the same extent improves the performance of the corresponding normalization using all probes. Therefore, to present comparable results only, we have separated results from these two types of subset-normalizations using knowledge about which genes are regulated. Thus, for the Golden Spike data set, we mainly compare BGX and PPLR with the analysis based on MAS5 expression indexes, and the results from RMA and GCRMA pre-processed data is mainly compared separately.

More complicated models often come with the prize of longer computer run times. Of the methods evaluated the BGX model and the PPLR method together with the multi-mgMOS model are the most computer intense ones. The computer run time for one single two group analysis of 3+3 HG-U95A arrays with data from 12626 genes is more than 24 hours with BGX and 1.5 hours for PPLR+multi-mgMOS (using the recommended EM method of PPLR) on a 2.2 GHz AMD Opteron machine. The corresponding time (including pre-processing of PM and MM data) is 2–3 minutes for PLW and 9 seconds for the moderated t-test in LIMMA.

## Conclusion

We have presented two new methods for ranking genes with respect to differential expression: Probe level Locally moderated Weighted median-t (PLW) and Locally Moderated Weighted-t (LMW). Both methods perform very well compared to existing methods with PLW having the most accurate ranking of regulated genes in four out of five examined spike-in data sets with RMA and GCRMA processed data. With LMW we show that introducing an intensity-level dependent scale parameter for the prior distribution of the gene-specific variances improves the performance of the moderated t-test. Also, compared to the moderated t-statistic, LMW shows a much more stable false positive rate across intensity-levels when used on MAS5 expression indexes. In the PLW method inference is performed directly on logged PM intensities and the median of the resulting moderated t-statistics for each probe-set is used to find differentially expressed genes. Overall the PLW method performs better than all compared methods and thus probe-level inference appears to be preferable over the standard approach using gene expression indexes for inference.

## Authors' contributions

MÅ provided the initial idea, formulated the model, derived and programmed the estimation procedure, erformed the analysis of real and simulated data, and drafted the manuscript. All authors finalized and approved the final version of the manuscript.

## Supplementary Material

Additional file 1 Parameter estimation details. A detailed description of the parameter estimation procedure. Click here for file

Additional file 2 R-code and package information. The R-code used for each of the methods compared together with information about used R-packages (names and versions). Click here for file

Additional file 3 ROC curves for all methods. Figures with ROC curves for all methods compared for each of the five data sets. Click here for file

Additional file 4 ROC curve AUC for all methods. Tables with ROC curve AUC up to 25, 50, 100, 200 false positives for all methods compared for each of the five data sets. Click here for file
