# Supplementary material for: Empirical Bayes models for multiple probe type microarrays at the probe level
Source: BMC Bioinformatics. 2008 Mar 20;9:156. doi: 10.1186/1471-2105-9-156 (PMC2358895; doi:10.1186/1471-2105-9-156)
Supplement: Additional file 1 — Parameter estimation details. A detailed description of the parameter estimation procedure. [file 1471-2105-9-156-S1.pdf]

Parameter estimation in PLW. Supplement to the article titled "Empirical Bayes models for multiple probe type arrays at the probe level".

Magnus Åstrand

October 1, 2007

We consider step 1: estimating the parameters m, β and Σ_A. Step 2 is follows directly as a special case of step 1.

Let x_p denote the $q-1$ sized sub-vectors obtained by dropping the last dimension of the vectors z_p and let $H_p = H(\bar{y}_p)$ where $H : \mathbb{R} \to \mathbb{R}^{2K-1}$ is a set B-spline basis functions for a given set of K interior spline-knots, see chapter 5 of Hastie et al. (2001). Thus for $p = 1, \dots, P$

$$\begin{aligned} x_p | c_p &\sim \mathrm{N}_{q-1}(0, c_p \Sigma_A) \\ c_p &\sim \Gamma^{-1}(\tfrac{1}{2}m, \tfrac{1}{2}m \cdot \exp\{H_p^T \beta\}) \end{aligned} \tag{1}$$

where $\mathrm{N}_n(\mu, \Sigma)$ denotes the n-dimensional normal distribution with mean μ and covariance matrix Σ, and $\Gamma^{-1}(k, \theta)$ is the inverse-gamma distribution with density function

$$f(x) = \frac{\theta^k}{\Gamma(k)} x^{-(k+1)} \exp\{-\theta/x\} \ , \quad x > 0 \ .$$

For later use, note that for $X \sim \Gamma^{-1}(k, \theta)$ we have $\mathrm{E}[\log(X)] = \log(\theta) - \psi(k)$ and $\mathrm{E}[X^{-1}] = k/\theta$ from the properties of the log-gamma and gamma distribution respectively (Johnson et al. 1995, page 89-90). Here $\psi(x) = \frac{d}{dx} \log \Gamma(x)$ is the digamma function.

Treating x_p and c_p as observed data the contribution from probe p to the full log-likelihood (ignoring constant terms) of model (1) is

$$\begin{aligned} \mathcal{L}_p &= -\frac{1}{2} \log(|\Sigma_A|) - \frac{x_p^T \Sigma_A^{-1} x_p}{2c_p} \\ &\quad + \frac{m}{2} H_p^T \beta - \frac{m \cdot \exp\{H_p^T \beta\}}{2c_p} \\ &\quad - \frac{m}{2} \log(c_p) + \frac{m}{2} \log(\frac{m}{2}) - \log(\Gamma(m/2)) \ . \end{aligned} \tag{2}$$

In the E-step of the EM-algorithm the expectation of $\mathcal{L}_p$ is calculated with respect to the conditional distribution of c_p given x_p and governed by the parameter estimates from the previous iteration: m_0, β_0 and Σ_{A0}:

$$Q_p = \mathrm{E}[\mathcal{L}_p | x_p, m_0, \beta_0, \Sigma_{A0}]$$

Since c_p given x_p is Γ^{-1}-distributed with parameters

$$\frac{m_0+q-1}{2} \quad \text{and} \quad \frac{x_p^T\Sigma_{A0}^{-1}x_p + m_0\exp\{H_p^T\beta_0\}}{2}$$

we have

$$\begin{aligned}
Q_p \;=\;& -\frac{1}{2}\log(|\Sigma_A|) - \frac{x_p^T\Sigma_A^{-1}x_p}{2}\cdot\frac{m_0+q-1}{x_p^T\Sigma_{A0}^{-1}x_p + m_0\exp\{H_p^T\beta_0\}} \\
& +\frac{m}{2}\Big(H_p^T\beta - \exp\{H_p^T\beta\}\cdot\frac{m_0+q-1}{x_p^T\Sigma_{A0}^{-1}x_p + m_0\exp\{H_p^T\beta_0\}}\Big) \\
& -\frac{m}{2}\left(\log\Big(\frac{x_p^T\Sigma_{A0}^{-1}x_p + m_0\exp\{H_p^T\beta_0\}}{2}\Big) - \psi\Big(\frac{m_0+q-1}{2}\Big)\right) \\
& +\frac{m}{2}\log(\frac{m}{2}) - \log(\Gamma(m/2)) \;. \qquad (3)
\end{aligned}$$

With

$$w_p = \frac{m_0+q-1}{x_p^T\Sigma_{A0}^{-1}x_p + m_0\exp\{H_p^T\beta_0\}} \;.$$

we can rewrite Q_p as

$$\begin{aligned}
Q_p \;=\;& -\frac{1}{2}\log(|\Sigma_A|) - \frac{x_p^T\Sigma_A^{-1}x_p}{2}\cdot w_p \\
& +\frac{m}{2}\Big(H_p^T\beta - \exp\{H_p^T\beta\}\cdot w_p\Big) \\
& +\frac{m}{2}\left(\log(w_p) + \log\Big(\frac{m}{m_0+q-1}\Big) + \psi\Big(\frac{m_0+q-1}{2}\Big)\right) \\
& -\log(\Gamma(m/2)) \;. \qquad (4)
\end{aligned}$$

In the M-step of the EM-algorithm $Q_1+\cdots+Q_P$ is maximized with respect to m, β, and Σ_A. Starting with Σ_A, since only the first row of (4) depends on Σ_A the updated estimate of Σ_A is obtained directly as

$$\hat{\Sigma}_A = \frac{1}{P}\sum_{p=1}^{P} w_p x_p x_p^T \;. \qquad (5)$$

Continuing with β only the second row of (4) needs to be considered. However, here numerical optimization is needed to find an updated estimate of β by maximizing the function $h(\beta)$ defined as

$$h(\beta) = \frac{1}{P}\sum_{p=1}^{P}\Big(H_p^T\beta - w_p\cdot\exp\{H_p^T\beta\}\Big)$$

with gradient equal to

$$\nabla h(\beta) = \frac{1}{P}\sum_{p=1}^{P} H_p\Big(1 - w_p\cdot\exp\{H_p^T\beta\}\Big) \;.$$

Since each evaluation of the function (and gradient) involves summing over all probes a quasi-Newton optimization method (variable metric algorithm) implemented in C is used for shorter computer run times. Given the updated estimate $\hat{\beta}$, we then find the value of m maximizing $Q_1 + \cdots + Q_P$ through numerical optimization of the function f:

$$f(m) = \frac{m}{2}\Big(\log(m) + S\Big) - \log\Big(\Gamma\big(m/2\big)\Big) \ .$$

where

$$S = h(\hat{\beta}) + \psi\Big(\frac{m_0 + q - 1}{2}\Big) - \log(m_0 + q - 1) + \frac{1}{P}\sum_{p=1}^{P} \log(w_p) \ . \qquad (6)$$

References

Hastie, T., R. Tibshirani, and J. Friedman (2001). *The Elements of Statistical Learning* (First ed.), Volume 1. Springer.

Johnson, N., S. Kotz, and N. Balakrishnan (1995). *Continuous Univariate Distributions* (Second ed.), Volume 2. John Wiley and Sons.
