# Supplementary material for: Empirical Bayes models for multiple probe type microarrays at the probe level
Source: BMC Bioinformatics. 2008 Mar 20;9:156. doi: 10.1186/1471-2105-9-156 (PMC2358895; doi:10.1186/1471-2105-9-156)

Figure S1: ROC curves based on RMA pre−processed data

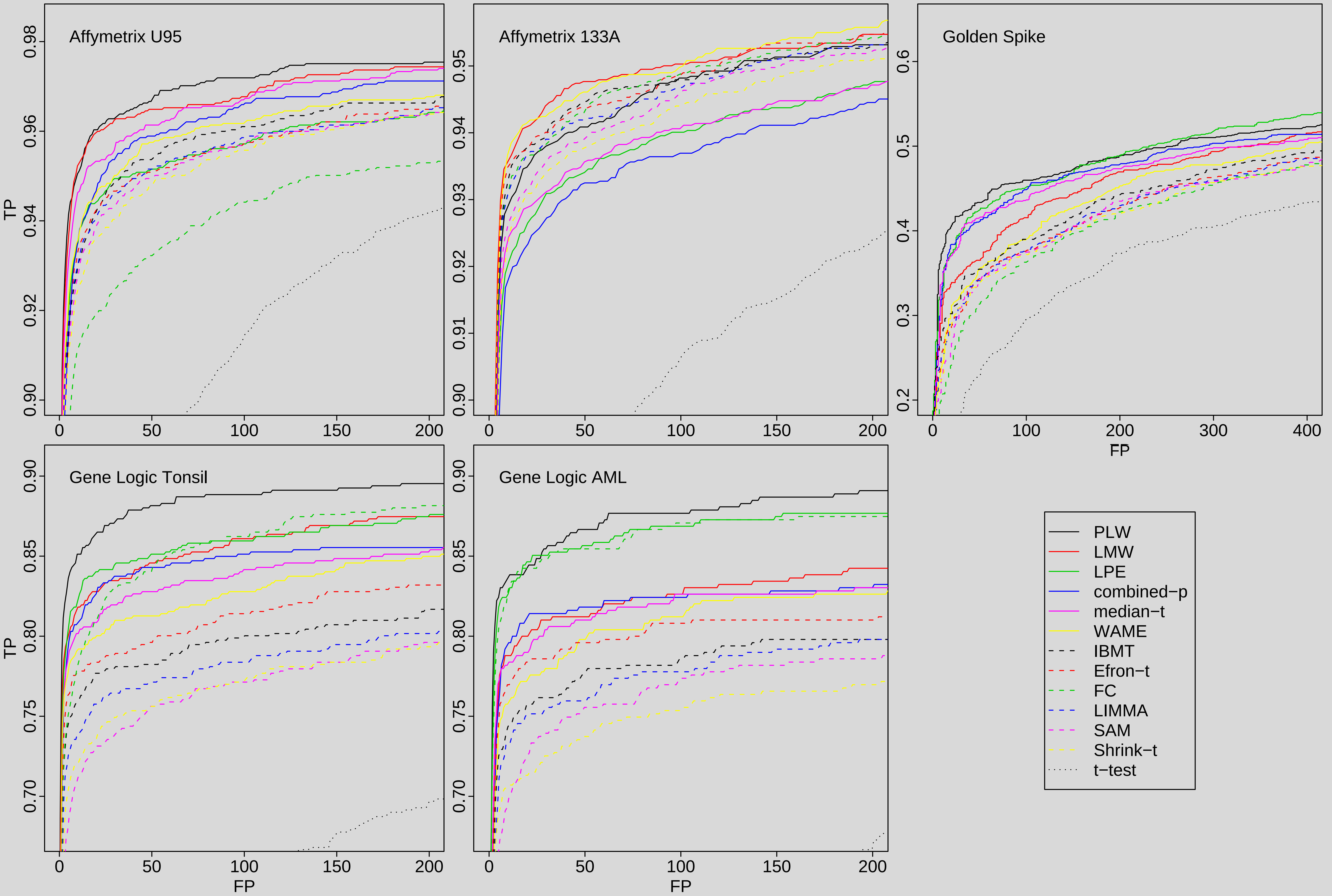

Figure S2: ROC curves based on GCRMA pre−processed data

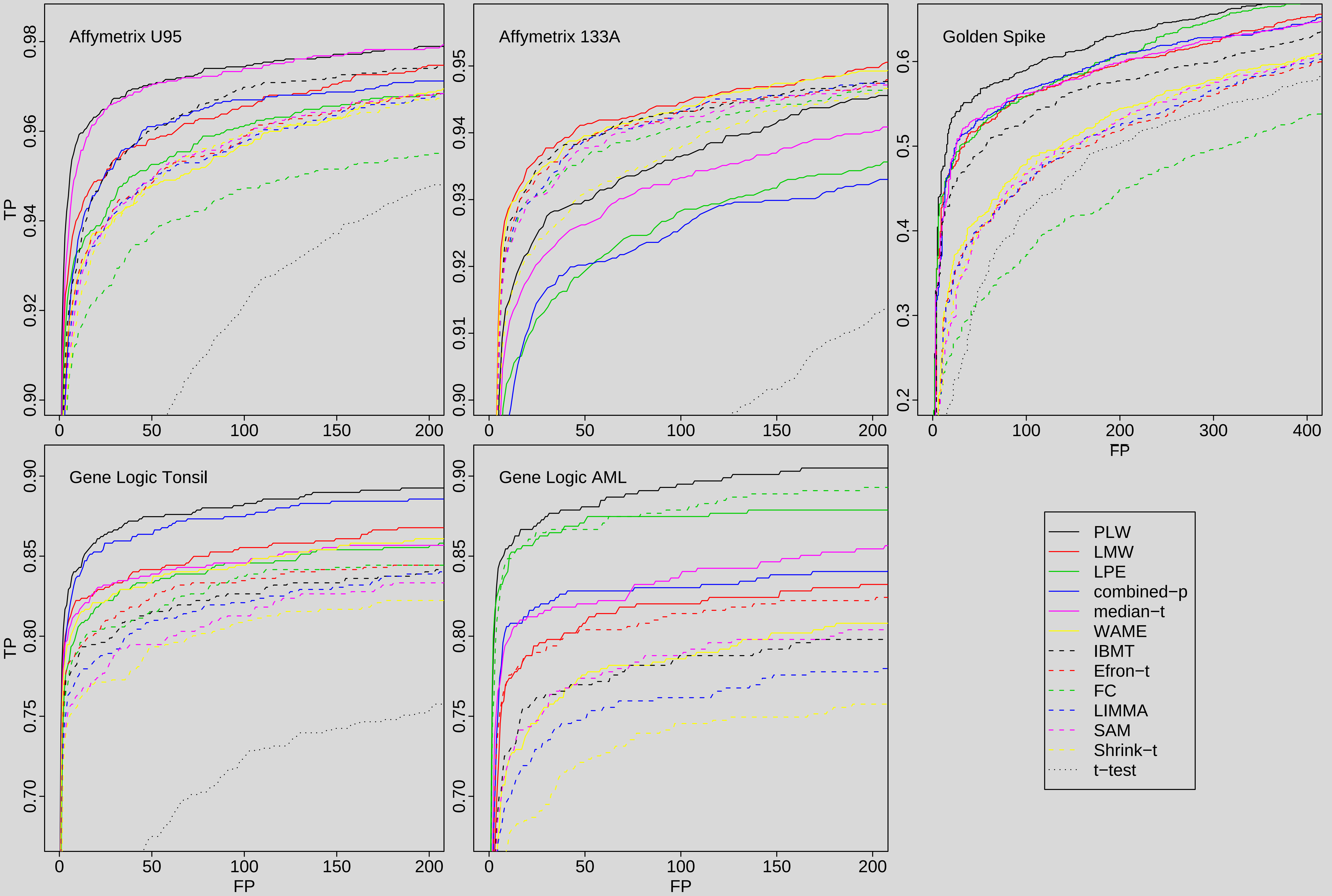

Figure S3: ROC curves based on MAS5 pre−processed data, together with ROC curves for PPLR, BGX, and logit−t
Note the range of y axis, line colors, and line types differs from Figures S1 and S2

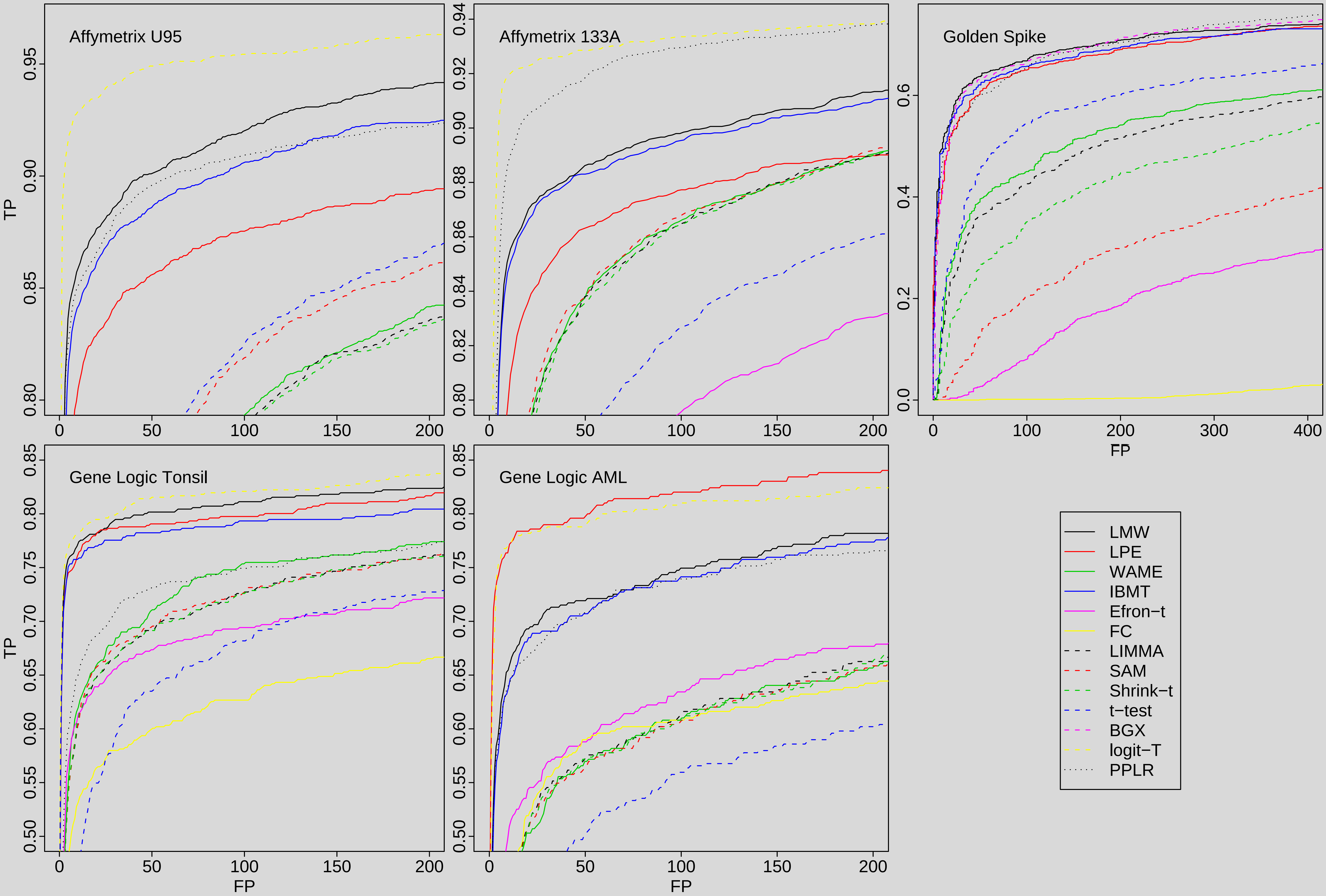

Supplement: Additional file 3 — ROC curves for all methods. Figures with ROC curves for all methods compared for each of the five data sets. [file 1471-2105-9-156-S3.pdf]
