# Supplementary material for: Empirical Bayes models for multiple probe type microarrays at the probe level
Source: BMC Bioinformatics. 2008 Mar 20;9:156. doi: 10.1186/1471-2105-9-156 (PMC2358895; doi:10.1186/1471-2105-9-156)
Supplement: Additional file 4 — ROC curve AUC for all methods. Tables with ROC curve AUC up to 25, 50, 100, 200 false positives for all methods compared for each of the five data sets. [file 1471-2105-9-156-S4.pdf]

Table 1: Area under ROC curves up to 25, 50, 100 and 200 false positives standardized so that optimum is 1000 and rounded to nearest integer value. Results are based on RMA pre-processed data. Numbers within parenthesis are within data set rank for the compared methods. Methods are ordered with respect to mean rank across data sets for AUC up to 100.

	Affymetrix U95A			
Method	25 FP	50 FP	100 FP	200 FP
PLW	940(1)	952(1)	961(1)	968(1)
LMW	939(2)	951(2)	958(2)	965(2)
LPE	920(5)	935(6)	945(7)	953(7)
combined-p	916(7)	936(5)	949(4)	963(3)
WAME	924(4)	938(4)	949(5)	959(4)
median-t	932(3)	945(3)	955(3)	957(5)
IBMT	913(9)	932(9)	945(6)	955(6)
Efron-t	918(6)	933(7)	944(8)	953(8)
FC	886(12)	907(12)	923(12)	936(12)
LIMMA	916(8)	932(8)	944(9)	953(9)
SAM	912(10)	929(10)	941(10)	951(10)
Shrink-t	908(11)	926(11)	939(11)	950(11)
t-test	746(13)	805(13)	852(13)	891(13)

	Affymetrix 133A			
Method	25 FP	50 FP	100 FP	200 FP
PLW	905(5)	922(5)	933(6)	942(6)
LMW	911(1)	928(1)	939(1)	946(1)
LPE	894(11)	913(11)	925(11)	935(11)
combined-p	890(12)	910(12)	922(12)	936(10)
WAME	908(2)	926(2)	937(2)	932(12)
median-t	899(8)	916(9)	927(10)	945(2)
IBMT	907(3)	925(3)	936(3)	943(4)
Efron-t	906(4)	924(4)	935(4)	944(3)
FC	902(7)	921(7)	934(5)	943(5)
LIMMA	903(6)	921(6)	933(7)	942(7)
SAM	898(9)	918(8)	930(8)	940(8)
Shrink-t	895(10)	916(10)	928(9)	938(9)
t-test	780(13)	826(13)	861(13)	888(13)

	Golden spike			
Method	25 FP	50 FP	100 FP	200 FP
PLW	349(1)	387(1)	419(1)	446(1)
LMW	289(5)	323(5)	359(5)	402(5)
LPE	326(2)	368(2)	404(2)	437(2)
combined-p	307(4)	354(4)	392(3)	426(4)
WAME	250(7)	292(7)	333(7)	429(3)
median-t	310(3)	357(3)	392(4)	379(6)
IBMT	269(6)	305(6)	339(6)	378(7)
Efron-t	248(8)	285(8)	324(8)	364(9)
FC	194(12)	243(12)	292(12)	343(12)
LIMMA	244(9)	284(9)	323(9)	364(8)
SAM	223(11)	272(11)	316(11)	360(10)
Shrink-t	242(10)	281(10)	319(10)	359(11)
t-test	108(13)	157(13)	210(13)	272(13)

	Gene Logic Tonsil			
Method	25 FP	50 FP	100 FP	200 FP
PLW	841(1)	859(1)	873(1)	882(1)
LMW	806(3)	823(3)	838(3)	853(3)
LPE	811(2)	828(2)	843(2)	855(2)
combined-p	795(4)	817(4)	832(4)	834(6)
WAME	778(6)	794(7)	807(7)	843(5)
median-t	789(5)	806(5)	820(6)	824(7)
IBMT	748(9)	764(9)	779(9)	793(9)
Efron-t	762(8)	776(8)	791(8)	808(8)
FC	767(7)	800(6)	828(5)	851(4)
LIMMA	727(10)	747(10)	763(10)	778(10)
SAM	692(12)	718(12)	741(12)	763(12)
Shrink-t	708(11)	730(11)	748(11)	766(11)
t-test	456(13)	513(13)	567(13)	621(13)

	Gene Logic AML			
Method	25 FP	50 FP	100 FP	200 FP
PLW	819(1)	839(1)	857(1)	871(1)
LMW	764(5)	788(5)	805(5)	820(4)
LPE	813(2)	833(2)	849(2)	862(2)
combined-p	765(4)	790(4)	806(4)	814(6)
WAME	734(8)	760(8)	783(8)	817(5)
median-t	761(6)	784(6)	801(6)	804(7)
IBMT	721(9)	744(9)	762(9)	779(9)
Efron-t	749(7)	770(7)	786(7)	798(8)
FC	808(3)	830(3)	846(3)	860(3)
LIMMA	712(10)	734(10)	754(10)	772(10)
SAM	677(12)	711(11)	737(11)	759(11)
Shrink-t	684(11)	706(12)	727(12)	746(12)
t-test	403(13)	465(13)	524(13)	580(13)

Table 2: Area under ROC curves up to 25, 50, 100 and 200 false positives standardized so that optimum is 1000 and rounded to nearest integer value. Results are based on GCRMA pre-processed data. Numbers within parenthesis are within data set rank for the compared methods. Methods are ordered with respect to mean rank across data sets for AUC up to 100.

	Affymetrix U95A			
Method	25 FP	50 FP	100 FP	200 FP
PLW	947(1)	958(1)	965(1)	971(1)
LMW	929(3)	942(3)	952(3)	961(4)
median-t	941(2)	954(2)	963(2)	970(2)
IBMT	920(5)	936(5)	950(5)	960(5)
combined-p	916(6)	934(6)	946(6)	956(6)
LPE	920(4)	938(4)	952(4)	962(3)
Efron-t	914(8)	929(7)	942(7)	953(7)
WAME	915(7)	929(8)	941(10)	952(10)
FC	895(12)	913(12)	928(12)	940(12)
LIMMA	913(9)	929(9)	941(9)	952(9)
SAM	911(10)	928(10)	941(8)	953(8)
Shrink-t	907(11)	925(11)	940(11)	952(11)
t-test	768(13)	821(13)	864(13)	900(13)

	Affymetrix 133A			
Method	25 FP	50 FP	100 FP	200 FP
PLW	891(8)	910(8)	922(8)	931(8)
LMW	901(1)	920(1)	931(1)	939(1)
median-t	882(10)	903(10)	917(10)	927(10)
IBMT	897(2)	893(12)	908(12)	919(12)
combined-p	868(12)	895(11)	910(11)	921(11)
LPE	875(11)	917(2)	929(2)	937(3)
Efron-t	894(4)	915(4)	928(4)	937(4)
WAME	896(3)	917(3)	929(3)	938(2)
FC	891(7)	912(7)	926(7)	935(7)
LIMMA	892(5)	914(5)	927(5)	936(5)
SAM	892(6)	913(6)	927(6)	936(6)
Shrink-t	883(9)	905(9)	920(9)	931(9)
t-test	748(13)	803(13)	843(13)	872(13)

	Golden spike			
Method	25 FP	50 FP	100 FP	200 FP
PLW	443(1)	497(1)	537(1)	575(1)
LMW	395(3)	451(4)	497(5)	538(5)
median-t	383(4)	453(3)	502(2)	541(4)
IBMT	365(6)	449(5)	498(4)	542(2)
combined-p	381(5)	458(2)	500(3)	542(3)
LPE	410(2)	421(6)	468(6)	514(6)
Efron-t	250(8)	316(8)	373(8)	433(10)
WAME	260(7)	328(7)	388(7)	450(7)
FC	205(12)	250(12)	297(13)	355(13)
LIMMA	246(9)	314(9)	372(9)	434(8)
SAM	219(11)	291(11)	363(11)	432(11)
Shrink-t	227(10)	298(10)	367(10)	434(9)
t-test	151(13)	212(13)	298(12)	383(12)

	Gene Logic Tonsil			
Method	25 FP	50 FP	100 FP	200 FP
PLW	836(1)	853(1)	866(1)	877(1)
LMW	809(3)	823(3)	836(3)	849(3)
median-t	807(4)	821(4)	832(4)	843(4)
IBMT	774(9)	842(2)	857(2)	870(2)
combined-p	822(2)	809(6)	825(6)	838(6)
LPE	789(6)	791(9)	806(9)	820(9)
Efron-t	780(7)	798(7)	815(7)	828(7)
WAME	798(5)	814(5)	827(5)	841(5)
FC	776(8)	792(8)	810(8)	826(8)
LIMMA	761(10)	780(10)	798(10)	814(10)
SAM	748(11)	770(11)	787(11)	807(11)
Shrink-t	745(12)	762(12)	781(12)	799(12)
t-test	528(13)	589(13)	644(13)	693(13)

	Gene Logic AML			
Method	25 FP	50 FP	100 FP	200 FP
PLW	837(1)	857(1)	873(1)	887(1)
LMW	738(7)	769(7)	793(6)	810(6)
median-t	774(4)	796(5)	812(5)	830(4)
IBMT	705(8)	797(4)	813(4)	825(5)
combined-p	770(5)	848(2)	861(2)	870(3)
LPE	830(2)	736(8)	757(8)	775(10)
Efron-t	749(6)	773(6)	790(7)	805(7)
WAME	690(10)	727(10)	754(10)	777(9)
FC	826(3)	846(3)	860(3)	874(2)
LIMMA	675(11)	708(11)	733(11)	753(11)
SAM	698(9)	731(9)	757(9)	777(8)
Shrink-t	642(12)	675(12)	705(12)	728(12)
t-test	431(13)	485(13)	530(13)	578(13)

Table 3: Area under ROC curves up to 25, 50, 100 and 200 false positives standardized so that optimum is 1000 and rounded to nearest integer value. Results are based on MAS5 expression indexes, (upper parts of sub-tables) together with results for BGX, logit-t, and PPLR (lower part of sub-tables). The last three all have their own pre-processing of PM and MM probe intensities, PPLR was applied to multi-mgMOS probe set summaries. Numbers within parenthesis are within data set rank for the compared methods, and methods are ordered with respect to mean rank across data sets for AUC up to 100 (Within MAS5 based results only). The results for BGX from the Gene Logic AML data set in *italic* is based on a subset of 1011 probe sets only.

	Affymetrix U95A			
Method	25 FP	50 FP	100 FP	200 FP
LMW	830(1)	862(1)	887(1)	910(1)
IBMT	810(2)	844(2)	870(2)	894(2)
LPE	762(3)	805(3)	836(3)	861(3)
WAME	587(5)	653(6)	712(6)	766(6)
SAM	610(4)	678(4)	737(4)	790(5)
LIMMA	584(7)	651(7)	708(7)	763(7)
Shrink-t	583(8)	649(8)	706(8)	760(8)
t-test	585(6)	665(5)	733(5)	791(4)
Efron-t	540(9)	599(9)	648(9)	690(9)
FC	454(10)	509(10)	556(10)	599(10)
BGX	-	-	-	-
logit-T	912	928	940	949
PPLR	826	857	880	899

	Affymetrix 133A			
Method	25 FP	50 FP	100 FP	200 FP
LMW	823(1)	851(1)	872(1)	889(1)
IBMT	818(2)	848(2)	869(2)	886(2)
LPE	774(3)	814(3)	843(3)	864(3)
WAME	699(5)	761(5)	808(5)	844(5)
SAM	708(4)	767(4)	812(4)	846(4)
LIMMA	694(6)	758(6)	805(6)	842(6)
Shrink-t	685(7)	753(7)	802(7)	840(7)
t-test	638(8)	702(8)	755(8)	801(8)
Efron-t	585(9)	666(9)	724(9)	769(9)
FC	460(10)	545(10)	608(10)	660(10)
BGX	-	-	-	-
logit-T	890	908	920	928
PPLR	853	883	905	919

	Golden spike			
Method	25 FP	50 FP	100 FP	200 FP
LMW	478(1)	549(1)	602(1)	647(1)
IBMT	463(2)	531(2)	586(2)	632(2)
LPE	434(3)	505(3)	569(3)	620(3)
WAME	165(5)	258(5)	342(5)	423(5)
SAM	17(8)	51(8)	110(8)	182(8)
LIMMA	143(6)	230(6)	311(6)	394(6)
Shrink-t	83(7)	150(7)	226(7)	314(7)
t-test	178(4)	284(4)	394(4)	485(4)
Efron-t	2(9)	8(9)	31(9)	88(9)
FC	0(10)	0(10)	1(10)	1(10)
BGX	414	513	582	636
logit-T	-	-	-	-
PPLR	486	572	635	688

	Gene Logic Tonsil			
Method	25 FP	50 FP	100 FP	200 FP
LMW	758(1)	777(1)	791(1)	805(1)
IBMT	745(3)	762(3)	775(3)	786(3)
LPE	749(2)	768(2)	781(2)	794(2)
WAME	591(4)	641(4)	688(4)	725(4)
SAM	583(6)	633(5)	673(5)	710(5)
LIMMA	579(8)	628(7)	670(6)	708(6)
Shrink-t	580(7)	629(6)	670(7)	708(7)
t-test	453(10)	533(10)	597(9)	654(9)
Efron-t	586(5)	624(8)	655(8)	681(8)
FC	509(9)	548(9)	582(10)	616(10)
BGX	-	-	-	-
logit-T	770	788	803	815
PPLR	628	673	707	734

	Gene Logic AML			
Method	25 FP	50 FP	100 FP	200 FP
LMW	636(2)	675(2)	703(2)	735(2)
IBMT	622(3)	660(3)	694(3)	726(3)
LPE	753(1)	773(1)	793(1)	812(1)
WAME	432(8)	490(8)	540(8)	588(7)
SAM	426(9)	487(9)	537(9)	586(9)
LIMMA	436(6)	496(6)	543(6)	591(5)
Shrink-t	432(7)	492(7)	540(7)	588(8)
t-test	326(10)	399(10)	466(10)	524(10)
Efron-t	481(4)	528(4)	571(4)	616(4)
FC	449(5)	508(5)	554(5)	590(6)
BGX	*604*	*695*	*750*	*790*
logit-T	748	768	785	800
PPLR	620	658	693	724
